# Atrophic Anterior Mandible Treated With Sandwich Osteotomy Without Mini‐Screws and Mini‐Plates: A Case Report With 7 Years of Follow‐Up

**DOI:** 10.1002/ccr3.70377

**Published:** 2025-04-09

**Authors:** Antonio Scarano, Ahmad G. A. Khater, Giovanni Falisi, Sergio Alexandre Gehrke, Sergio Rexhep Tari

**Affiliations:** ^1^ Department of Innovative Technologies in Medicine and Dentistry University of Chieti–Pescara Chieti Italy; ^2^ Faculty of Dental Medicine and Oral Health Sciences McGill University Montreal Quebec Canada; ^3^ Department of Life Health and Environmental Sciences University of L'Aquila L'Aquila Italy; ^4^ Department of Research Bioface/PgO/UCAM Montevideo Uruguay

**Keywords:** alveolar bone grafting, alveolar bone loss, biomaterials, bone regeneration, dental implant, mouth rehabilitation

## Abstract

Restoring the anterior mandible may be challenging due to both insufficient height and width of the edentulous alveolar ridge; thus, this case report aimed to treat anterior mandibular atrophy by using the inlay technique without the use of mini‐screws or mini‐plates to stabilize the augmented bone fragments. A 19‐year‐old patient who lost his anterior teeth in an accident was treated with a horizontal osteotomy performed 4 mm from the alveolar ridge, with two oblique cuts made using an ultrasonic instrument, and the final phase of the osteotomy was performed with a lever for dental extraction. One mini‐block of equine bone was inserted between the coronal osteotomized segment and the mandibular basal bone, with cancellous equine bone particles filling the residual space. A resorbable collagen membrane was used to cover the biomaterials and mini‐block. Seven days after the augmentation procedure, there were no signs of dehiscence, lesions, infection, or segment movement. Four months after surgery, a CBCT radiograph was obtained for implant placement, revealing a 5–7 mm vertical increase without bone resorption or height loss. The radiographic assessment showed a mineralized zone between basal bone and coronal portion of osteotomized segments, whereas the histological analysis showed new bone and osteoid matrix around and inside the block material. As a result, this case report indicated that using an equine collagenated block in alveolar bone augmentation resulted in high stability while eliminating the need for mini‐screws and mini‐plates, resulting in a simplified sandwich technique.

## 1 Introduction

Alveolar bone loss in the maxilla and the mandible makes placing dental implants more challenging, complicating the oral surgical rehabilitation of edentulous patients [1]. Tooth loss, periodontal diseases, inappropriate orthodontic treatment, trauma, and infection could all result in both vertical and horizontal bone loss. Hence, the resorbed alveolar ridge needs to be augmented with bone before dental implants are placed. Different regenerative approaches are currently being used to achieve adequate bone volume for the predictable placement of endosseous implants. As a result, many surgical techniques have been developed, including autogenous bone grafts, guided bone regeneration (GBR), alloplastic materials [2, 3, 4, 5], alveolar distraction osteogenesis, onlay bone grafting, and, most recently, the inlay approach [6].

In 1991, Dahlin and colleagues introduced GBR in dentistry [7]. They used expanded polytetrafluoroethylene membranes for bone regeneration and proposed them for posterior mandibular reconstruction, which has been used with high success rates [8, 9]. However, onlay autologous or allograft for vertical bone regeneration in posterior mandibles has shown a low success rate [10]. Furthermore, vertical augmentation is a highly delicate procedure that requires strict adherence to surgical protocol [11]. Titanium mesh, allografts, and autogenous bone grafts have all been successfully used for vertical ridge augmentation in atrophic jaws [12, 13]. However, infection is a common negative consequence of using titanium mesh and screws, which could lead to the loss of grafted bone and procedure failure.

In 1975, Franz Härle initially established the use of interpositional bone to increase height in the atrophic edentulous mandible [14]. This technique entails performing an alveolar osteotomy and moving the crestal bone according to the visor principle [14]. Subsequently, D Schettler and PJ Stoelinga later proposed combining the visor‐osteotomy and sandwich techniques to augment the atrophic edentulous mandible; thus, the autologous bone block graft is placed between osteotomized bony segments [15, 16]. However, this technique results in donor site morbidity [17]. Therefore, this case report aimed to describe how to treat local defects in the mandibular incisor area using a sandwich osteotomy and an interposition xenograft without using mini‐screws or mini‐plates to stabilize moving bone fragments.

## 2 Study Design and Informed Consent

This study follows the SCARE guidelines for reporting surgical case reports [18] and was conducted under the 1964 Helsinki Declaration and its later amendments or comparable ethical standards [19]. The involved patient provided written informed consent under the Helsinki Declaration for experimentation on human subjects [20].

## 3 Patient Information

### 3.1 Demographic Details and Presentation

A 19‐year‐old male patient with atrophy in the anterior mandible requested implant rehabilitation and was referred to the University of Chieti–Pescara's Oral Surgery Department for fixed prosthetic rehabilitation of the mandibular incisor region.

### 3.2 Patient's History

**FIGURE 1:**
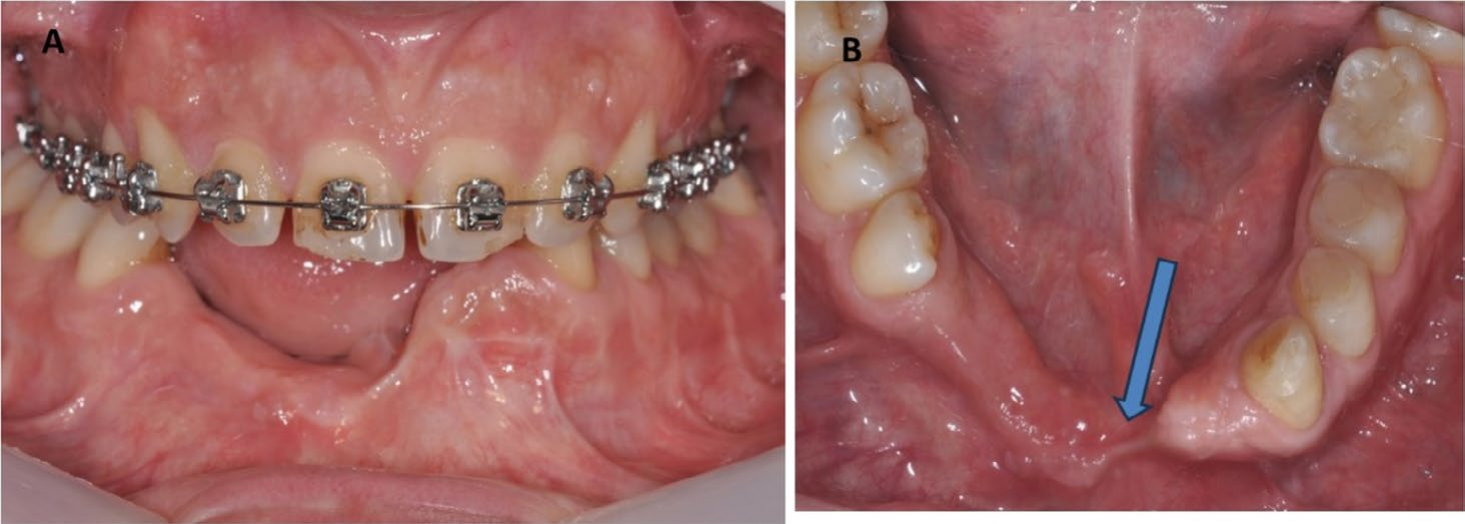
(A) Before vertical ridge augmentation of anterior mandibular alveolar crest, missing four incisors and right canine with a severely resorbed anterior mandible. (B) In the left area, also an insufficient thickness was present (Arrow).

The patient was physically healthy and did not smoke; sadly, he suffered a car accident that resulted in the loss of his mandibular lower incisors and right side canine teeth (Figure 1). He has good oral hygiene, a physiological periodontal probing, and only small areas of tooth decalcification. However, the patient refused removable prostheses, prosthetic bridges, and autogenous bone harvesting, instead emphasizing his preference for fixed prosthetic rehabilitation.

## 4 Diagnostic Assessment and Interpretation

Initially, the patient underwent a thorough clinical examination, which included extensive extra and intra‐oral examinations and a radiographic examination using cone‐beam computer tomography (CBCT). The clinical assessment also involved evaluating occlusion and inter‐arch distance. However, the CBCT radiographic evaluation revealed insufficient height in the mandibular incisor region, making it unsuitable for implant placement.

## 5 Clinical Findings

Given the patient's preference and condition of unsuitability for implant placement, an inlay procedure was proposed using a block of collagenated cancellous equine bone (Sp‐Block, OsteoBiol by Tecnoss, Coazze, Italy) to allow subsequent implant placement for prosthetic rehabilitation of the affected region of the mandible [21, 22].

## 6 Intervention

Before starting the surgical procedures, the patient rinsed his mouth for 1 min with 0.2% chlorhexidine digluconate solution (Curaden Healthcare S.p.A., Saronno VA, Italy). The procedures were then performed under local anesthesia using Articaine plus 1:100,000 epinephrine (Ubistesin 4% forte—3M ESPE, Dental AG Seefeld, Germany) and light intravenous sedation (Midazolam 2 mg/mL—Martindale Pharma, Buckinghamshire, UK).

**FIGURE 2:**
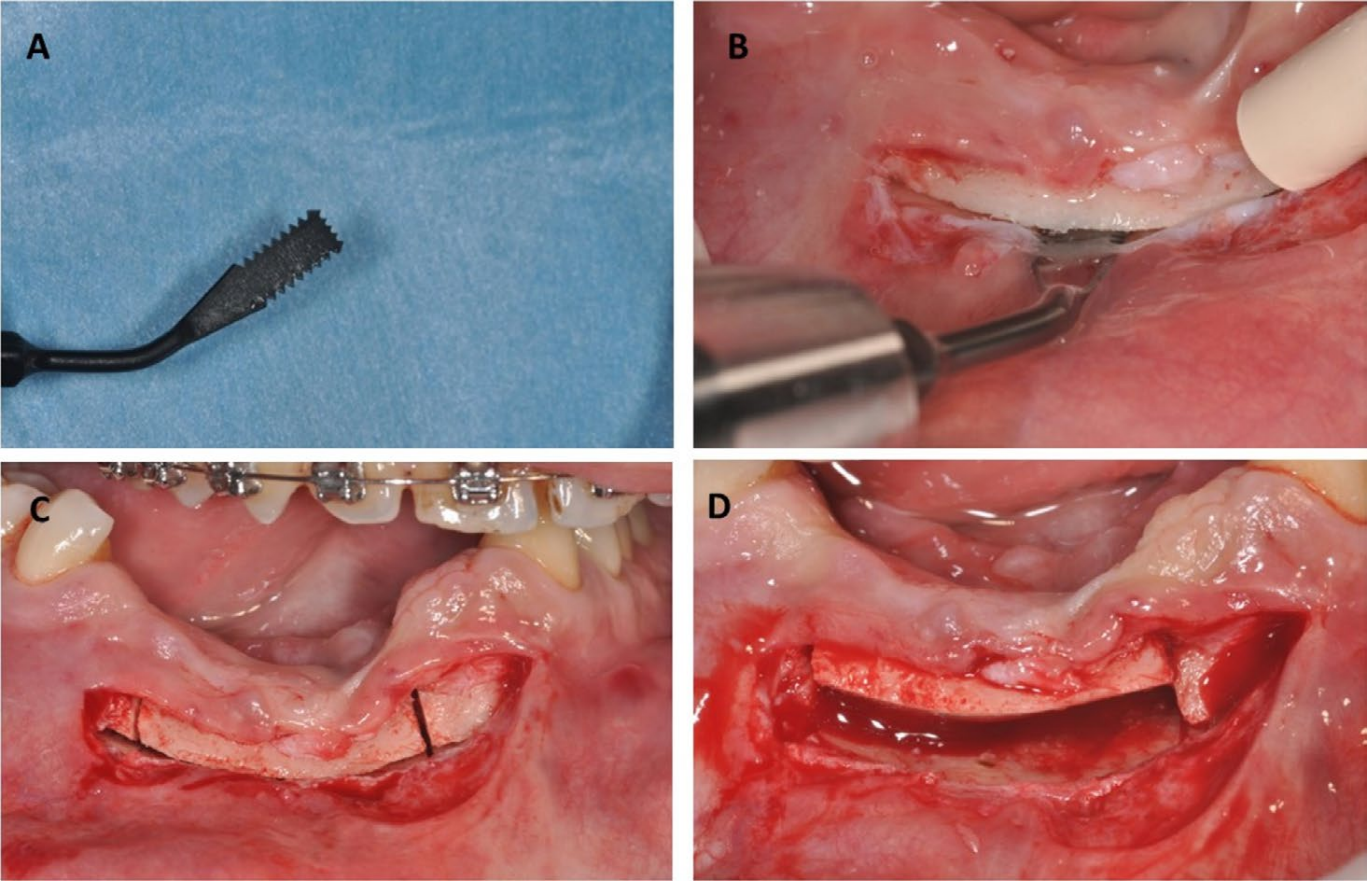
(A) A flat chisel serrated on three sides for osteotomies. (B) A basal osteotomy followed (C) two vertical osteotomies with the ultrasonic device. (D) The bone segment was moved superiorly after completing all bone cuts.

**FIGURE 3:**
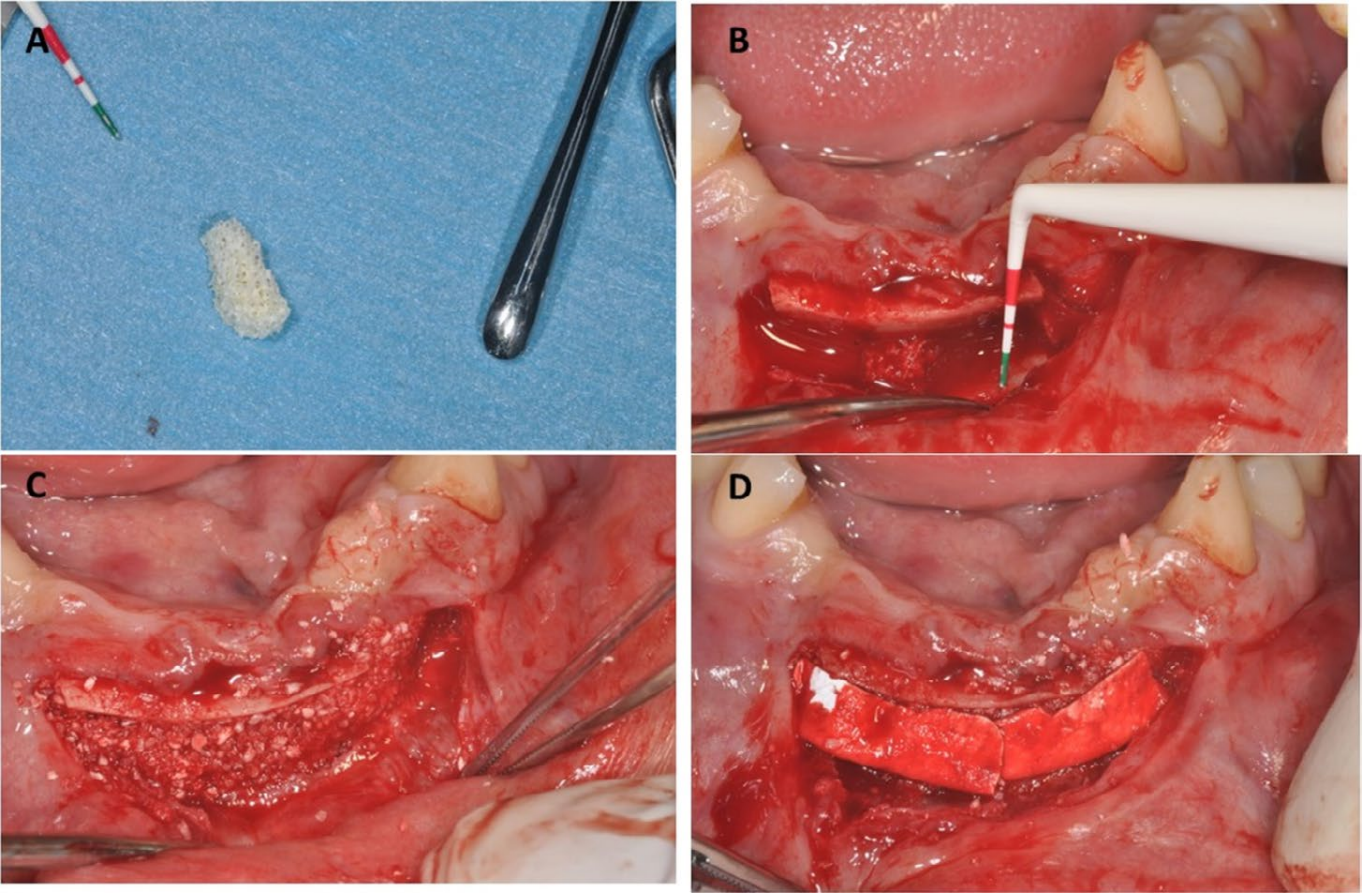
(A) Block of collagenated equine. (B) One block of collagenated equine bone interposed between the basal bone and the mobilized fragment. (C) Particles of cortical‐cancellous porcine bone filled the residual space. (D) A collagen membrane was used to cover the bone graft.

To preserve the mental nerve, a paracrestal incision between mandibular canines was made in the buccal vestibule, 3 mm below the mucogingival line (Figures 2 and 3), with subperiosteal tissue dissection limited to the buccal side. The soft tissues were carefully sectioned and managed to create a tension‐free environment for the graft. This included a single horizontal incision after the mucogingival line that allowed adequate tension‐free coverage of the grafting area.

**FIGURE 4:**
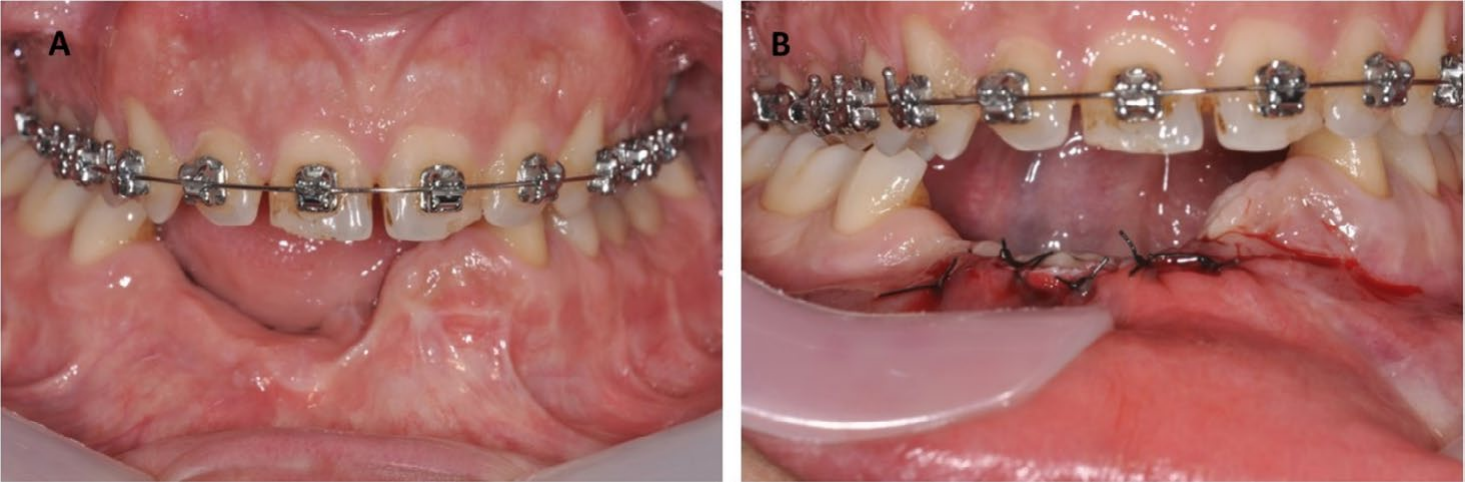
(A) Before and immediately after the regenerative procedure. (B) No periosteal releasing incisions were performed, and the flap was sutured.

**FIGURE 5:**
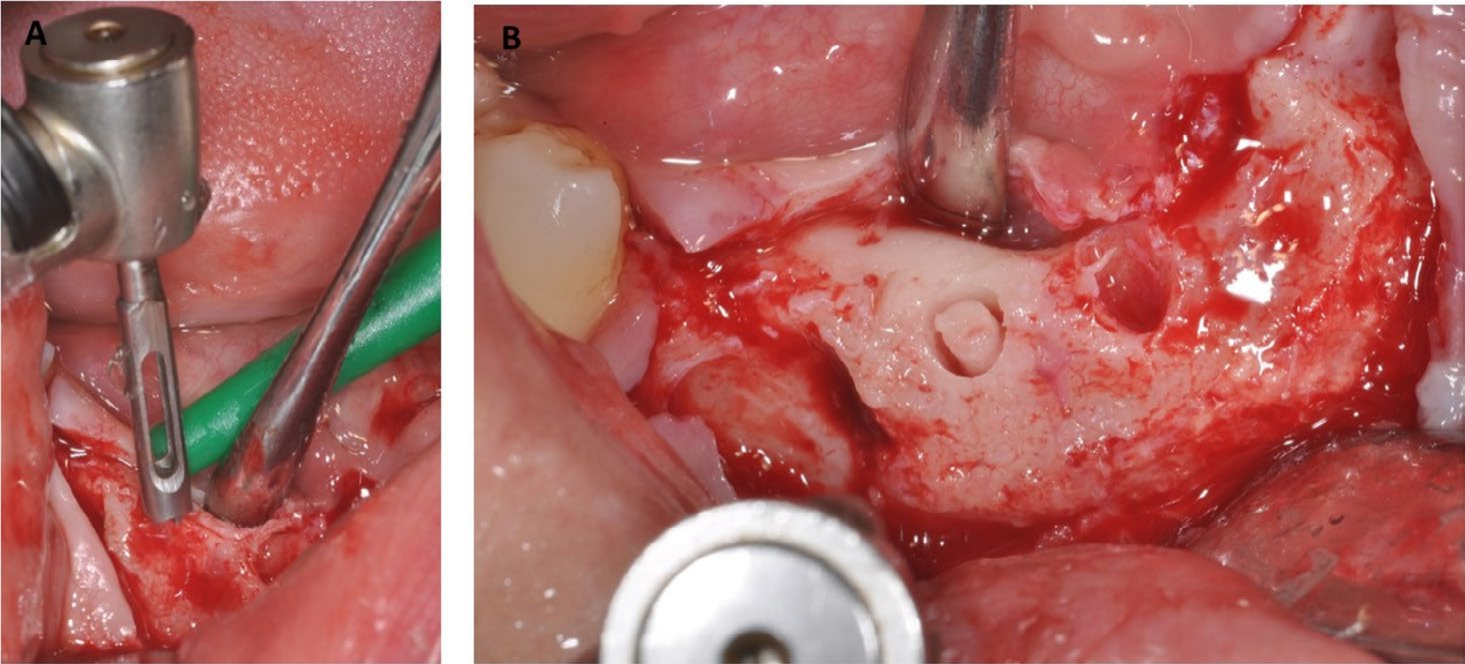
(A) After 6 months, a bone biopsy was retrieved with a trephine bur, and four implants were placed. (B) Clinically, a mature bone was present.

Thus, the full‐thickness flap was retracted to avoid tension around the mental nerve. To achieve an osteotomized segment height of at least 3 mm, a horizontal osteotomy was performed 2–3 mm above the alveolar ridge, followed by making two oblique cuts with an ultrasonic device (Surgysonic II—Esacrom S.R.L., Imola BO, Italy), leaving at least 1–2 mm distal to the left canine tooth and right premolar tooth (Figures 4 and 5). Such osteotomies were performed under cold (4°C–5°C) sterile saline irrigation, using a tip T‐Blak n°ES009ST (Figure 2a) and power 45, vibration 80, and pump capacity 100.

The lingual mucosa attached to the periosteum was left adherent to the osteotomized segment, and the final phase of the osteotomy was performed.

The osteotomized segment was then lifted coronally with a lever, which was applied between the basal bone and the osteotomized segment by imparting a tilting motion. Precise and complete cuts during osteotomy help reduce the risk of fracture.

At this stage, much attention was given to preserving the lingual periosteum. The bone block was shaped and contoured to fit the defect site precisely. This involved trimming and smoothing the edges to ensure a snug fit and reduce the risk of soft tissue perforation. Two mini‐blocks (5 × 5 × 3 mm) of equine bone (Sp‐Block, OsteoBiol by Tecnoss, Coazze, Italy) were then inserted between the coronal osteotomized segment and the mandibular incisor basal bone (Figure 3). The residual space was filled with cortical‐cancellous porcine bone particles (Gen‐Os, OsteoBiol by Tecnoss, Coazze, Italy), with no periosteal releasing incisions. The flap was then carefully sutured with Vicryl 4.0 (Ethicon FS‐2—Johnson & Johnson Medical N.V., Belgium).

## 7 Follow‐Up

The patients were clinically monitored every week for the first month following surgery and twice in the succeeding months before implant placement. The healing process was uneventful, with no neurosensory disturbances observed. Radiographic assessments were performed immediately after the surgical procedure using CBCT (Gendex GXDP‐700 Panorex + Cone‐Beam CBCT X‐ray, Chamblee, GA, USA) with exposure parameters of 110 KVp, 8 mA, and a 5 × 8 field of view (FOV) and a very low dosage.

Seven days after the regeneration procedure, there was no sign of dehiscence, lesions, infection, or segment movement; moreover, the alveolar ridge increased by 5.3 mm immediately throughout the first and third months after the bone augmentation surgery. Four months after surgery, a CBCT radiograph was obtained before placing implants, revealing a 5–7 mm vertical increase. Furthermore, CBCT assessments of the alveolar ridge at 4 months and 1 year following surgery revealed no bone resorption or height loss.

After these 4 months, a full‐thickness crestal flap was raised, and the soft tissue around the regenerated alveolar process was elevated. Then, a trephine bur (3 mm internal diameter and 13 mm length) was used for bone specimens obtained (Figure 5). The three biopsies were immediately collected in 10% formalin solution, and thin slices were obtained; the tissues were then processed using the Precise 1 Automated System (Assing, Rome, Italy). Afterward, four submerged Close BL implants (4 mm × 13 mm) with a screw‐retained conical abutment connection (Isomed, Due Carrare—PD, Italy) were placed in positions #32, #31, #41, and #43. The flap was meticulously sutured with Polimid 4.0 (Assut, Magliano de' Marsi, Italy) and removed after 6 days. Healing screws were inserted 4 months after the implants were placed, and 10 days afterward, impressions were taken. A preliminary prosthesis was used, followed by a permanent one with a metal‐ceramic crown after 1 month.

**FIGURE 6:**
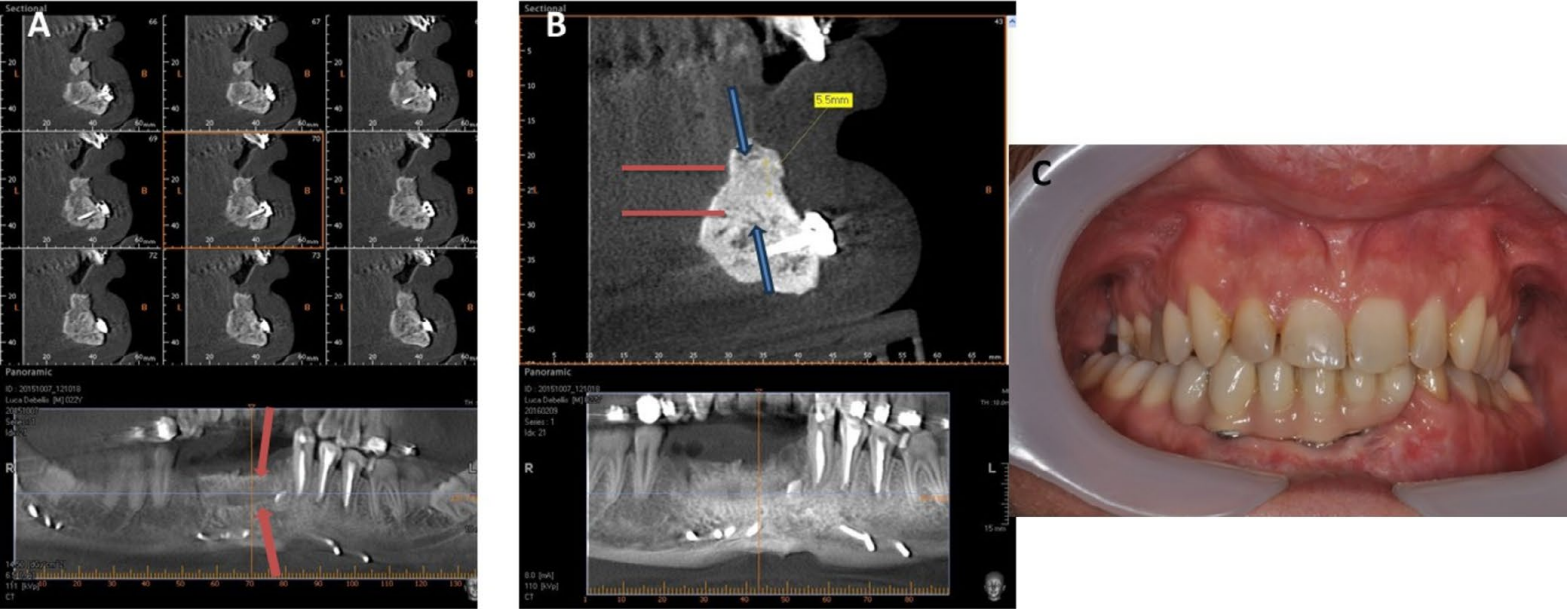
After 6 months, the CBCT scan showed bone gain. Four implants were placed. This is the final prosthetic outcome.

**FIGURE 7:**
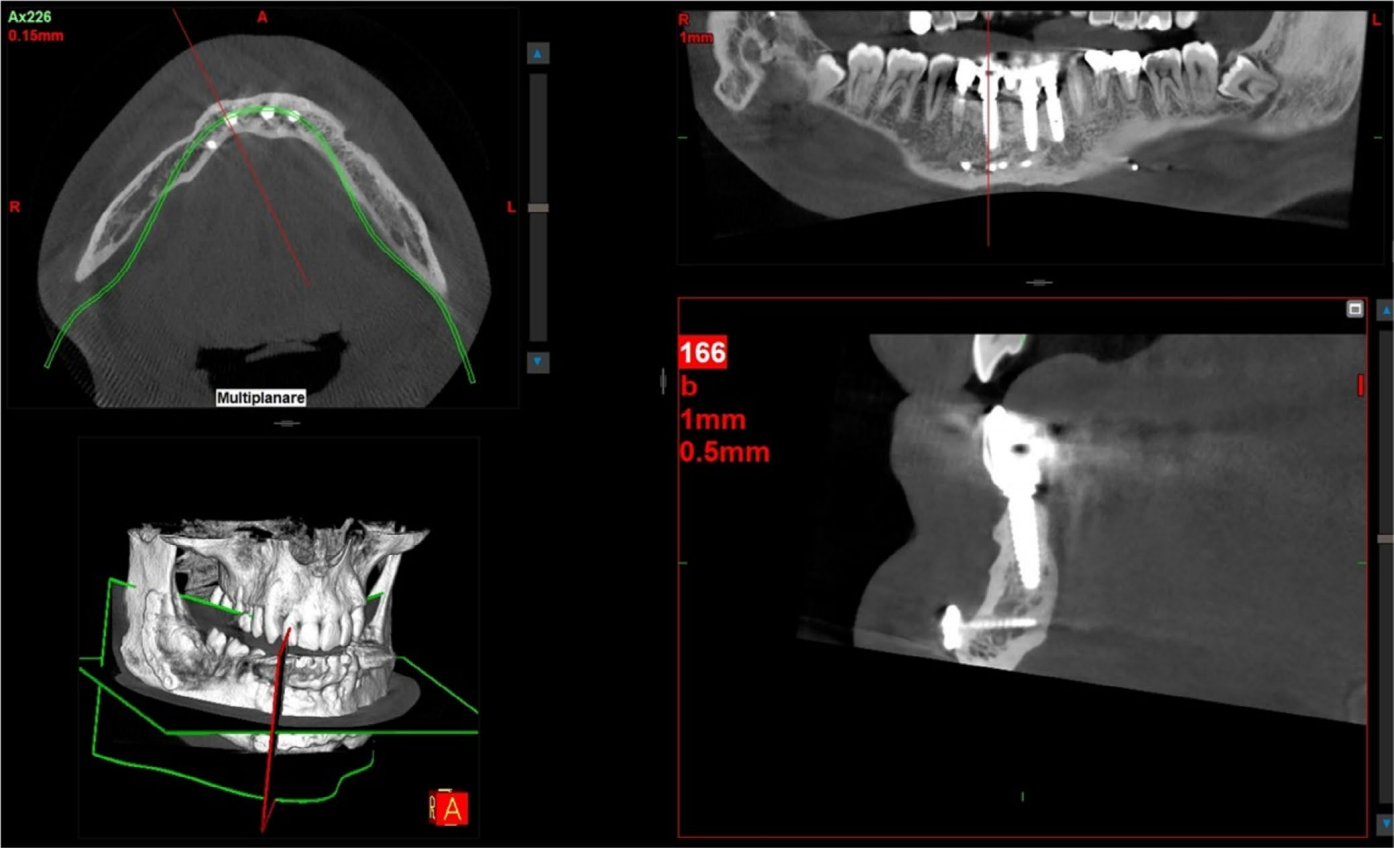
After 7 years, a CBCT scan showed bone gain and an absence of bone loss. This is a clinical aspect of prosthetic outcomes after 7 years.

After three and 7 years, a follow‐up of additional crestal bone assessment was done using CBCT, revealing that the crestal bone level remained almost constant. Also, there were no clinical signs of mucositis or peri‐implantitis (Figures 6 and 7).

## 8 Outcomes

**FIGURE 8:**
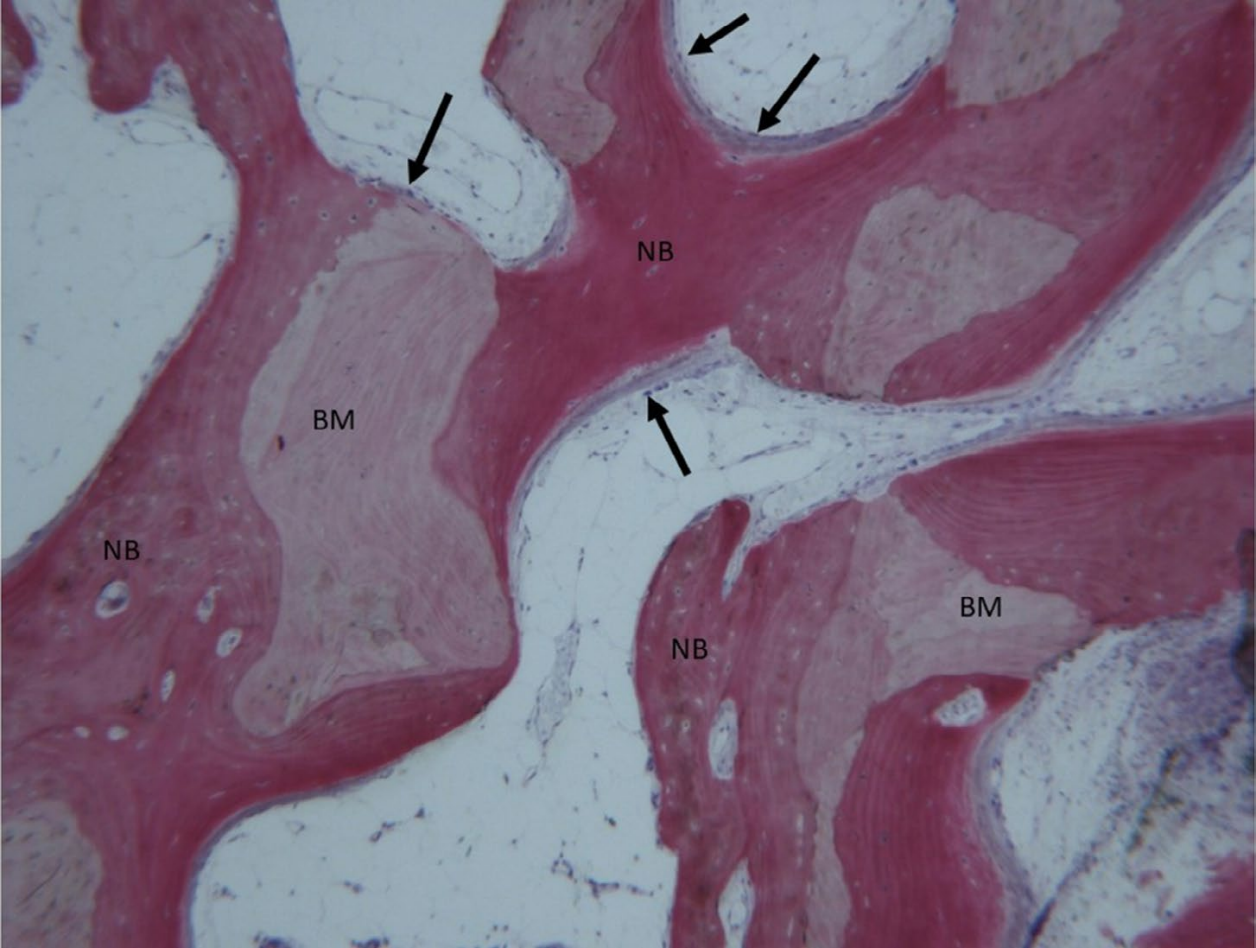
New bone (NB) and osteoid matrix were observed around and inside of the block material (BM). The periphery and central portion of the cavities showed mineralized new bone formation. The osteoid matrix was actively secreted by osteoblasts (arrows), and moderate numbers of marrow stromal cells and vascular networks contained in marrow spaces were observed. Acid fuchsine and toluidine blue, original magnification 50×.

Trabecular mature bone was apparent at low magnification; however, young bone and osteoid matrix were only noticeable around and inside the block material (Figure 8). All specimens showed no pathogenic inflammatory cell infiltration (e.g., neutrophils, macrophages, etc.), epithelial cells, connective tissue, or foreign body response, and the block material was surrounded by new bone. Also, the specimens indicated conspicuous woven and mature bone; however, mature bone derived from the endosteal surface occupied the external portion of the bone sinus, whereas mineralized new bone formation occurred on the periphery and central portions of the cavities. Furthermore, the osteoid matrix actively secreted by osteoblasts and moderate numbers of marrow stromal cells and vascular network contained in marrow spaces were observed, notably multiple osteoblasts and unmineralized matrix with collagen fibrils in areas of new bone apposition. As such, the tissues in the sample comprised 4.9% ± 1.9% lamellar bone, 46% ± 1% woven bone, and 38% ± 3.8% marrow spaces.

## 9 Discussion

In this case report, anterior mandibular atrophy was effectively treated with interposition sandwich bone grafts, without the use of mini‐screws or mini‐plates. Such effectiveness was evidenced by a post operative follow‐up without any adverse events and a high level of graft integration, as noted during radiographic follow‐up. The piezosurgery device simplified the technique and reduced the incidence of complications [23, 24, 25].

The recently revised inlay approach makes implant placement easier by raising the bone above the nerve and improving interocclusal distance and crown‐implant ratios. However, many clinical problems have been recorded following and during bone grafting, including cortical bone fractures, membrane exposure, bone resorption, and neurological impairments [26]. Still, the lack of micromovement and the blood supply are essential for successfully integrating grafted biomaterials and substituting new bone [7]. For instance, studies by Barone et al. and Felice et al. revealed a high success rate with the inlay graft approach for treating posterior mandible atrophy [27, 28]. Furthermore, a recent meta‐analysis found that inlay techniques using bone substitute material were effective for increasing vertical alveolar ridge height in the atrophic posterior mandible before implant placement, as well as reported decreased peri‐implant marginal bone loss and high implant survival, without significant differences between autogenous bone block grafts and bone substitutes [29].

Several researchers have already used the interposition inlay bone grafting technique with a fixation device [27, 28]; however, few researchers have used it without a fixation device, which has the advantage of a lower risk of failure and complications such as fracture due to the usage of mini‐screws and mini‐plates. Vertical augmentation and inlay techniques are critical in the reconstruction of alveolar ridges, particularly in cases of severe bone loss due to trauma, periodontitis, or extraction. Different studies have compared the efficacy of onlay and inlay grafting techniques for vertical ridge augmentation. In a randomized clinical trial, the inlay technique with simultaneous implant placement showed a mean vertical bone gain of 3.34 mm compared to −0.02 mm in the onlay group, indicating a significant advantage for the inlay technique [30]. Another study comparing onlay symphysis cortico‐cancellous bone block with the sandwich (inlay) technique found no statistically significant differences in the percentage of newly formed bone, although the onlay technique required less time [31]. A systematic review highlighted that both techniques are stable for at least 4–5 years, with the onlay technique showing more marginal bone level change after the first year of loading [32]. A recent systematic review found that the mean vertical bone gain for the GBR technique was 4.7 mm, with less resorption compared to the onlay block graft [33].

Based on our findings, bone regeneration of vertical deficiencies in the anterior mandible using an inlay approach without mini‐screws and mini‐plates seems feasible and predictable, enabling successful implant placement and osseointegration.

## 10 Conclusion

In conclusion, this case report showed that using an equine collagenated block as an inlay bone graft resulted in high stability, eliminating the need for mini‐screws and mini‐plates by using a simplified sandwich technique. However, further research is needed to support these findings.

## Author Contributions

**Antonio Scarano:** project administration, validation, writing – original draft. **Ahmad G. A. Khater:** conceptualization, data curation, writing – review and editing. **Giovanni Falisi:** conceptualization, supervision, writing – review and editing. **Sergio Alexandre Gehrke:** conceptualization, data curation, resources, writing – review and editing. **Sergio Rexhep Tari:** software, writing – review and editing.

## Ethics Statement

The authors have nothing to report.

## Consent

Written informed consent was obtained from the patient to publish this report in accordance with the journal's patient consent policy.

## Conflicts of Interest

The authors declare no conflicts of interest.

## Data Availability

The data that support the findings of this study are available in the manuscript.
